# Editorial: Nutrients Recycling in Hydroponics: Opportunities and Challenges Toward Sustainable Crop Production Under Controlled Environment Agriculture

**DOI:** 10.3389/fpls.2022.845472

**Published:** 2022-03-14

**Authors:** Md Asaduzzaman, Genhua Niu, Toshiki Asao

**Affiliations:** ^1^Olericulture Division, Horticulture Research Center, Bangladesh Agricultural Research Institute, Gazipur, Bangladesh; ^2^Texas A&M AgriLife Research, Texas A&M University, Dallas, TX, United States; ^3^Faculty of Life and Environmental Science, Shimane University, Matsue, Japan

**Keywords:** nutrient recycling, plant factories, soilless culture, close-loop crop cascade, fruit qualities, wastewater reuse (WR), aquaponics

## Nutrient Recycling in Hydroponics

Hydroponics is a method of growing plants in soilless media or nutrient solution containing all essential mineral nutrients that potentially lead to yield and quality improvement (Gruda, 2009). In principle, nutrient solutions used in hydroponics can either be reused or discarded (Jensen, 1997; Nederhoff and Stanghellini, 2010). Nowadays, cultivation of horticultural crops including leafy and fruiting vegetables and medicinal herbs with pharmaceutical value are commercially grown in recycled (i.e., recirculating) hydroponics under controlled environments (Resh, 2012; Son et al., 2020).

In recycled hydroponics, nutrient solutions passed through the growing medium are collected into a reservoir and reused repeatedly. In this system, both water and mineral nutrients are used efficiently, therefore minimizing wastage of fertilizer and pollution of the environment. This type of hydroponic system has been widely used in controlled environment agriculture facilities including plant factories using artificial grow lights such as light-emitting diodes. Management of the hydroponic nutrient solution composition, along with the adjustment of environmental conditions may enhance the desired nutritional quality of the produce, regulate bioactive compounds, and increase antioxidants and other phytochemical content in soilless culture (Savvas et al., 2013; Asaduzzaman et al., 2018; Ciriello et al., 2021).

Hydroponics may however be challenged by the accumulation of root exudates that affect plant growth and reduce crop yield and quality. Lower growth and yield performance of several crops including lettuce, strawberry, several leafy vegetables, and ornamentals have been reported in recycled hydroponics (Asao et al., 2004, 2007; Lee et al., 2006). Reduced growth and yield of crops grown in recycled hydroponics because of increased concentration of phytotoxic root exudates have been reported causing allelochemical stress in the strawberry (Kitazawa et al., 2005), tomato (Yu and Matsui, 1993), and cucumber (Yu and Matsui, 1994). Certain phytotoxic chemicals may have a significant impact on plant growth. Moreover, recycled nutrient solutions usually require sterilization to minimize pathogen loads. In this regard, chemicals and physical treatments such as hypochlorite, ozone, and UV-light, are being used for sterilization.

The present Research Topic collected 12 scientific contributions from the leading research groups throughout the world working on recycled hydroponics, nutrient solution management, the influence of LEDs on crop growth and physiology, salinity impact on growth and nutritional quality, wastewater based nutrient recycling, and also nutrient reclamation or re-mineralization. In addition, this Research Topic complies several aspects of controlled environment agriculture that are useful with for scientific community, and by extension workers and commercial entrepreneurs, for the understanding of nutrient recycling in hydroponics toward sustainable crop production. The original research and reviewed literature also present how the techniques of nutrient recycling, efficient use of available nutrients, quality improvement of crop produce, and nutrient recycling from renewable resources will help the development of sustainable agricultural systems.

## Nutrient Solution Management in Recycled Hydroponics

Recycled hydroponics gained popularity in controlled environment agriculture leading to efficient use of costly fertilizer and a sustainable environment. Rufí-Salís et al. proposed a cascade system with a long-cycle tomato donor crop and five successive cycles of lettuce for a rooftop greenhouse. They quantified the scale between the donor and receiving crops and proposed three major ideas to optimize the nutrient flows while maintaining the yield and quality of the vegetables produced in the receiving crop. The variation of the nutrient content of the leachates produced by the donor crop was a key consideration that determines the number of plants to be planted as the receiving crop. It was found that the early stage of the donor crop could only produce 0.1 lettuces per tomato plant, with N as the limiting nutrient. On the other hand, the late stage of the donor crop was able to leach enough nutrients to feed 9 lettuces per tomato plant. However, attention must be paid to the electrical conductivity (EC) of the water flow to stay within non-harmful values. Nevertheless, the cascade system was shown to be efficient to mitigate the nutrient discharge of open systems, especially in terms of N and P to avoid eutrophication impacts in the early stage of the tomato crop. Considering the nutritional problems at the beginning of the cycle of the donor crop and the harmful salinity that can be reached at the end, future research needs to be designed to test different kinds of horticultural crops to discover possible viable combinations of donor and receiving crops.

Miller et al. reported that nutrient deficiencies in hydroponic production can also be observed due to recycling nutrient solutions. They evaluated the effects of recycling on solution EC changes, tissue nutrient concentration, canopy growth rate, plant water status, and shoot and root weight of lettuce in a greenhouse and suggest the development of optimal strategies for managing recycling nutrient solution in hydroponic production. This study indicates that continuous recycling with tap water containing moderate to high levels of alkalinity can result in an apparent increase in solution EC, nutrient deficiencies in the plants, and reduction in shoot growth, in spite of maintaining the solution EC at a target level. Results of this study also indicated that nutrient recycling significantly decreased N, P, K, and Fe and increased Na and Cu levels in the tissue, in addition to increasing solution EC between adjustments compared to the control. Through image analysis of plants reveals the negative effects of recycling on canopy area started 2 weeks after transplanting. Thus, they hypothesized that certain unwanted compounds (e.g., bicarbonates) and slowly consumed elements (e.g., Ca, Mg) were added to the recycling solution through the alkaline tap water with time.

Ahn et al. designed an EC-based nutrient recycling soilless culture system by theoretical and experimental analyses. An integrated model of solutes such as K^+^, Ca^2+^, and Mg^2+^ and water transport in growing media, automated nutrient solution preparation, and nutrient uptake was designed. In the simulation, the intrinsic characteristics of nutrient changes among open-, semi- closed-, and closed-loop soilless cultures were compared, and stochastic simulations for nutrient control were performed in the closed-loop system. Four automated irrigation modules for comparing nutrient changes among the soilless culture systems were constructed for sweet pepper grown in the greenhouse. Theoretical and experimental analyses exhibited that nutrient variations in these culture systems can be integrated as a function of nutrient supply to the system's boundary areas. Furthermore, stochastic simulation analysis indicated that the nutrient ratio in the soilless culture system reveals the nutrient uptake parameter-based deterministic patterns. Thus, they suggested that the nutrient ratio in the closed-loop soilless culture could be controlled by the long-term feedback of this ratio.

The quality of crop produce can be improved through quantitative management of the hydroponic nutrient solution. The desired mineral content in fruits and vegetables can be either increased or decreased through their elevated or deficient concentration in the culture solution. Zhu et al. studied appropriate ${\text{NH}}_{4}^{+}$/${\text{NO}}_{3}^{-}$ ratio that triggers plant growth and nutrient uptake of flowering Chinese cabbage by optimizing the pH value of the nutrient solution. They analyzed the changes in nutrient solution composition, the content of different N forms in plant tissues and exudates, and expression of plasma membrane H^+^-ATPase genes under different ${\text{NH}}_{4}^{+}$/${\text{NO}}_{3}^{-}$ ratios (0/100, 10/90, 25/75, 50/50). Compared with the control, ${\text{NH}}_{4}^{+}$/${\text{NO}}_{3}^{-}$ ratios (0/100, 10/90, and 25/75) significantly reduced the ${\text{NO}}_{3}^{-}$ content and increased the ${\text{NH}}_{4}^{+}$, amino acid, and soluble protein contents of flowering Chinese cabbage to varying extents. ${\text{NH}}_{4}^{+}$/${\text{NO}}_{3}^{-}$ ratio (10/90) significantly increased the N use efficiency, whereas ${\text{NH}}_{4}^{+}$/${\text{NO}}_{3}^{-}$ ratio (25/75) significantly decreased it to about 70.25% of that control. Owing to the difference in N absorption and utilization among seedlings, the pH value of the nutrient solution differed under different ${\text{NH}}_{4}^{+}$/NO_3_ ratios.

## Led Lighting, Nitrogen Metabolism and Environmental Controls in Recycled Hydroponics

In a plant factory with artificial lighting (PFAL), LEDs have widely been used for economic considerations and also to ensure a lower amount of heat emission inside the controlled room. The influence of LED spectrum along with the application of amino acid as nitrogen source has been studied by Talukder et al. (2018). Li et al. studied the effect of the LED spectrum on the quality and nitrogen metabolism under recycled hydroponics. They found that LED illumination spectra had a significant influence on the growth and nitrogen metabolism of lettuce. Adding green, purple, and far-red light had a negative impact on lettuce growth through decreased photosynthetic photon flux density. Purple LED supplementation was found to be conducive to vitamin-C accumulation in lettuce leaves. Adding purple light inhibited NR (nitrate reductase) and NiR (nitrite reductase) activities and caused a low nitrate, nitrite, and ammonium content while they contributed to amino acid accumulation for nitrogen assimilation. Thus, red, blue, and purple LEDs are recommended for use as supplemental lighting strategy greenhouse production.

He et al. grew purslane (*Portulaca oleracea* L.) in different NaCl salinities in hydroponics under LED lightings. Greater shoot and root dry mass with higher proline and carotene concentration were observed under 100 mM NaCl than fresh water, 200 and 300 mM. However, increasing salinity levels such as 200 and 300 mM NaCl decreases the shoot and root dry mass, ascorbic acid, and total phenolic compounds under lower leaf water content and photosynthetic performance. They concluded that it is feasible to grow purslane under 100 mM NaCl to achieve higher productivity and better quality.

In controlled environment agriculture, environmental factors including VPD fluctuation greatly influence the photosynthesis and yield of a plant. Inoue et al. examined the effects of the vapor pressure deficit (VPD) fluctuation on the photosynthetic and growth parameters in lettuce. In this study, gas exchange, chlorophyll fluorescence, and biomass accumulation were evaluated under drastic (1.63 kPa for 6 min and 0.63 for 3 min) or moderate (1.32 kPa for 7 min and 0.86 kPa for 3 min) VPD fluctuation. The drastic VPD fluctuation induced a gradual decrease in stomatal conductance and thus CO_2_ assimilation rate during the measurements, while moderate VPD fluctuation caused no reduction of these parameters. Thus, moderate VPD fluctuation maintained leaf expansion and the efficiency of CO_2_ diffusion across the leaf surface, resulting in enhanced plant growth compared with drastic VPD fluctuation.

## Organic Nutrient Supplementation Through Wastewater Treatment

Organic nutrients supplemented through wastewater treatment has great advantages in plant growth and yield and environmental sustainability. Researchers are applying a number of methods on the recovery of nutrients from wastewater and agricultural waste for organic greenhouse production (Voogt et al., 2011). Kechasov et al. have designed a closed hydroponic system with an integrated nitrification bioreactor. They compared plant development, fruit yield, and quality of tomatoes grown with the liquid by-product of biogas production from pig manure with greenhouse tomatoes grown with mineral fertilizers. The tomatoes grown with the organic waste-based fertilizer had a similar yield but poorer taste characteristics when compared with tomatoes grown with the high-mineral fertilizer. The plants grown with the organic waste-based treatment accumulated a higher amount of salts, especially tissue Cl^−^ content. Fertilizers based on organic wastes change plant development toward a generative state and can partially recover the physiological and biochemical responses seen in plants grown under suboptimal fertilization conditions, suggesting that these fertilizers could be favored over mineral fertilizers with similar inorganic compositions. However, the use of organic waste-based fertilizers is less feasible than high-mineral fertilizers because of the lower quality of tomato fruits produced.

Chow et al. verified the viability of nickel electroplating industrial wastewater effluent diluted at different concentrations as a source of nutrient recycling using a hydroponic soilless cultivation system. The significant inhibition of the root and shoot elongation and reduction of photosynthetic pigments were accompanied by the profound morphological distortions in the xylem, phloem, and stomata. It was observed that beyond the maximum tolerable concentration level at 25% of wastewater effluent for hyacinth bean and 5% of wastewater effluent for pak choi. The accumulation of proline and upregulation of POD and APX activities were detected against the nickel electroplating industrial wastewater-induced oxidative stress injury in the plant models.

Celletti et al. investigated the possibility of using innovative fertilizer solutions in hydroponic systems for the growth of agricultural plants. Aqueous hydrothermal carbonized liquid (AHL) derived from cow manure digestate was chemically characterized for pH, electrical conductivity, mineral elements, and organic compounds. The AHL diluted with distilled water (1:30, 1:60, and 1:90, v/v) was used as a source of nutrients instead of standard hydroponic nutrient solution and bio-assayed using maize plants. The results indicated that the dilution ratio 1:30 of the AHL solution showed higher phytotoxicity while the increased dilutions (1:60 and 1:90) had a lower level of toxicity allowing plants to grow. It was clearly evident that higher dilution ratios contain insufficient essential nutrients for the plant, showing pronounced leaf chlorosis. Further studies recommended are identifying appropriate species-specific dilution ratios to supply both low levels of phytotoxins and adequate content of essential nutrients for appropriate plant growth and development.

## Sustainable Crop Production Through Nutrient Recycling in Aquaponics

Cifuentes-Torres et al. mentioned that reclaimed water can, in theory, be used in aquaponics as it has been used as a water source in agriculture irrigation and aquaculture for many decades. They highlighted that there is an opportunity to use reclaimed water in aquaponics although there are still many questions that arise and more studies are needed to demonstrate that this technology is sustainable. There is the potential that toxic compounds such as certain toxic metals at low concentrations can function as food supplies in fish diets, under strict and controlled conditions. The presence of microalgae in aquaponic systems can be an advantage as it acts as both a food producer and wastewater treatment process. This Research Topic emphasized the studies with aquatic organisms and plants with the ability to metabolize contaminants without the risk to human health.

Lobanov et al. reported challenges of closed environment agriculture for resource-use optimization. The exploitation of readily available, soluble aquaculture effluent would be imperative for nutrient transfer in the hydroponic environment considering the role of microorganisms and the rhizosphere. In this regard, nutrient re-mineralization has to be adopted due to the challenges and carbon reduction and the additional costs associated with existing waste revalorization systems. They investigated micronutrient profiles of the re-mineralized effluent, traditional coupled aquaponics, and commercial hydroponic nutrient solutions were measured. Nutrient concentrations were significantly lower in the aquaculture-derived treatments than the commercial solution, while plant sap analysis did not follow the evidence of higher nutrient content in lettuce grown under excessive nutrient conditions. Lettuce grown in the commercial hydroponic nutrient solution likewise experienced deficiencies in Mg and Ca (young leaves) as well as Na and Si (both young and old leaves). The uptake of certain elements (Cu, Fe, Mg, S, and Zn) was greater across aquaponic treatments than initially predicted, however, Mn was universally absent from aquaponic treatments. B and P were especially low in the standard aquaponics treatment, i.e., fertilization with soluble RAS nutrients only. Together this suggests that the solids treatment system in parallel to RAS soluble effluent may be advantageous for aquaponic facilities seeking to maximize the benefits of the fish solids for plant nutrition. Nonetheless, iron remains the most capricious element to provide for plants. The evidence that neither the commercial solution nor aquaponic treatments were wholly successful in increasing iron uptake suggests a need for future studies to determine minimal “optimal” concentrations for plants and as well the real repercussions of deficiencies on crop yield and nutritional quality.

## Implications and Future Challenges of Recycled Hydroponics

Recycled hydroponics has great implications in practice under controlled environment agriculture toward economic considerations and environmental sustainability. It is generally used in greenhouses and indoor farming plant factories for producing a range of high-value crops such as leafy and fruiting vegetables and medicinal plants under artificial light organized vertically. Nutrient recycling is important to achieve high resource use efficiency. Simple EC control of nutrient solution concentration would be sufficient for growing vegetables sustainably under recycled hydroponics (Bamsey et al., 2012; Jung et al., 2015; Chowdhury et al., 2021). As EC indicates the total ionic balance of solution, the specific mineral requirement of the plant is usually overlooked. In this regard, ion-selective electrodes (ISEs) have been used for improving plant growth and quality with efficient use of major nutrients (Rius-Ruiz et al., 2014; Cho et al., 2017, 2018; Chowdhury et al., 2020). Therefore, the profitability of hydroponic farming can be achieved through yield maximization under recycled hydroponics based on either EC- or ISE- control of nutrient solution management.

The main concern of recycled hydroponics is the occurrence of pathogens due to the recirculating nature of the culture solution. Therefore, appropriate sterilization is essential for recycled hydroponics. Another important challenge of recycled hydroponics is the accumulation of inhibitory allelochemicals causing yield and quality reduction due to the autotoxicity phenomenon. Although a number of methods have been suggested to overcome the autotoxicity in several crops more appropriate recovery strategies are the future research need.

## Author Contributions

MA, GN, and TA have made a substantial, direct, and intellectual contribution to the work, and approved the editorial for publication in Frontiers in Plant Science. MA prepared the original manuscript. GN and TA reviewed and edited revised manuscript.

## Conflict of Interest

The authors declare that the research was conducted in the absence of any commercial or financial relationships that could be construed as a potential conflict of interest.

## Publisher's Note

All claims expressed in this article are solely those of the authors and do not necessarily represent those of their affiliated organizations, or those of the publisher, the editors and the reviewers. Any product that may be evaluated in this article, or claim that may be made by its manufacturer, is not guaranteed or endorsed by the publisher.

## References

[B1] Asaduzzaman M. Talukder M. R. Tanaka H. Ueno M. Kawaguchi M. Yano S.. (2018). Production of low-potassium content melon through hydroponic nutrient management using perlite substrate. Front. Plant Sci. 9:1382. 10.3389/fpls.2018.01382 30283488 PMC6157450

[B2] Asao T. Kitazawa H. Ban T. Pramanik M. H. R. (2004). Search of autotoxic substances in some leaf vegetables. J. Japanese Soc. Hort. Sci. 73, 247–249. 10.2503/jjshs.73.247

[B3] Asao T. Kitazawa H. Ushio K. Sueda Y. Ban T. Pramanik M. H. R. (2007). Autotoxicity in some ornamentals with means to overcome it. HortSci. 42, 1346–1350. 10.21273/HORTSCI.42.6.1346

[B4] Bamsey M. Graham T. Thompson C. Berinstain A. Scott A. Dixon M. (2012). Ion-specific nutrient management in closed systems: the necessity for ion-selective sensors in terrestrial and space based agriculture and water management systems. Sensors 12, 13349–13392. 10.3390/s121013349 23201999 PMC3545570

[B5] Cho W. J. Kim H. J. Jung D. H. Kang C. I. Choi G. L. Son J. E. (2017). An embedded system for automated hydroponic nutrient solution management. Trans. ASABE 60, 1083–1096. 10.13031/trans.12163

[B6] Cho W. J. Kim H. J. Jung D. H. Kim D. W. Ahn T. I. Son J. E. (2018). On-site ion monitoring system for precision hydroponic nutrient management. Comput. Electron. Agric. 146, 51–58. 10.1016/j.compag.2018.01.019

[B7] Chowdhury M. Islam M. N. Reza M. N. Ali M. Rasool K. Kiraga S.. (2021). Sensor-based nutrient recirculation for aeroponic lettuce cultivation. J. Biosyst. Eng. 46, 81–92. 10.1007/s42853-021-00089-8

[B8] Chowdhury M. Jang B. E. Kabir M. S. N. Kim Y. J. Na K. D. Park S. B.. (2020). Factors affecting the accuracy and precision of ion-selective electrodes for hydroponic nutrient supply systems. Acta Hortic. 1296, 997–1004. 10.17660/ActaHortic.2020.1296.126

[B9] Ciriello M. Formisano L. Pannico A. El-Nakhel C. Fascella G. Duri L. G.. (2021). Nutrient solution deprivation as a tool to improve hydroponics sustainability: yield, physiological, and qualitative response of lettuce. Agron 11,1469. 10.3390/agronomy11081469

[B10] Gruda N. (2009). Do soil-less culture systems have an influence on product quality of vegetables? J. Appl. Bot. Food Qual. 82, 141–147. 10.18452/9433

[B11] Jensen M. H. (1997). Hydroponics. HortSci. 32, 1018–1021.

[B12] Jung D. H. Kim H. J. Choi G. L. Ahn T. I. Son J. E. Sudduth K. A. (2015). Automated lettuce nutrient solution management using an array of ion-selective electrodes. Trans. ASABE 58, 1309–1319. 10.13031/trans.58.11228

[B13] Kitazawa H. Asao T. Ban T. Pramanik M. H. R. Hosoki T. (2005). Autotoxicity of root exudates from strawberry in hydroponic culture. J. Hortic. Sci. Biotech. 80, 677–680. 10.2503/hortj.UTD-R009

[B14] Lee J. G. Lee B. Y. Lee H. J. (2006). Accumulation of phytotoxic organic acids in reused nutrient solution during hydroponic cultivation of lettuce (*Lactuca sativa* L.). Sci. Hortic. 110, 119–128. 10.1016/j.scienta.2006.06.013

[B15] Nederhoff E. Stanghellini C. (2010). Water use efficiency of tomatoes. Pract. Hydrop. Greenhouses 115,52. Available online at: https://edepot.wur.nl/156932

[B16] Resh H. M. (2012). Hydroponic Food Production: A Definitive Guidebook for the Advanced Home Gardener and the Commercial Hydroponic Grower. Boca Raton, FL: CRC Press.

[B17] Rius-Ruiz F. X. Andrade F. J. Riu J. Rius F. X. (2014). Computer-operated analytical platform for the determination of nutrients in hydroponic systems. Food Chem. 147, 92–97. 10.1016/j.foodchem.2013.09.114 24206690

[B18] Savvas D. Gianquinto G. Tuzel Y. Gruda N. (2013). “Soilless culture,” in Good Agricultural Practices Principles for Greenhouse Vegetable Production in the Mediterranean Region (FAO Paper), 303–354.

[B19] Son J. E. Kim H. J. Ahn T. I. (2020). “Hydroponic systems,” in: *Plant Factory: An Indoor Vertical Farming System for Efficient Quality Food Production, 2nd Edn*, eds T. Kozai, G. Niu, and M. Takagaki (Cambridge, MA: Academic Press), 273–283. 10.1016/B978-0-12-816691-8.00020-0

[B20] Talukder M. R. Asaduzzaman M. Tanaka H. Asao T. (2018). Light-emitting diodes and exogenous amino acids application improve growth and yield of strawberry plants cultivated in recycled hydroponics. Sci. Hortic. 239, 93–103. 10.1016/j.scienta.2018.05.033

[B21] Voogt W. de Visser P. H. E. van Winkel A. Cuijpers W. J. M. van de Burgt G. J. H. M. (2011). Nutrient management in organic greenhouse production: navigation between constraints. Acta Hortic. 915, 75–82. 10.17660/ActaHortic.2011.915.9

[B22] Yu J. Q. Matsui Y. (1993). Extraction and identification of phytotoxic substances accumulated in nutrient solution for the hydroponic culture of tomato. Soil Sci. Plant Nutr. 39, 691–700. 10.1080/00380768.1993.10419186

[B23] Yu J. Q. Matsui Y. (1994). Phytotoxic substances in root exudates of cucumber (*Cucumis sativus* L.). J. Chem. Ecol. 20, 21–31. 10.1007/bf02065988 24241696

